# Comparison of Speech, Spatial, and Qualities of Hearing Scale (SSQ) and the Abbreviated Profile of Hearing Aid Benefit (APHAB) Questionnaires in a Large Cohort of Self-Reported Normal-Hearing Adult Listeners †

**DOI:** 10.3390/audiolres13010014

**Published:** 2023-02-10

**Authors:** Nirmal Srinivasan, Sadie O’Neill

**Affiliations:** Department of Speech-Language Pathology and Audiology, Towson University, Towson, MD 21252, USA

**Keywords:** SSQ, APHAB, aging, self-reported normal hearing

## Abstract

The Speech, Spatial, and Qualities of Hearing Scale (SSQ) and the Abbreviated Profile of Hearing Aid Benefit (APHAB) are two most commonly used questionnaires in the audiology clinic to assess an individual’s self-perception of their hearing ability. Here, we present the outcomes of these two questionnaires on a large group of self-reported normal hearing adult listeners. A total of 254 self-reported normal-hearing younger and older adults completed the SSQ and the APHAB questionnaire. The younger participants completed the questionnaires through Qualtrics, whereas the older participants completed the questionnaire through Qualtrics and a traditional pen-and-paper method. The younger listeners perceived a higher ability compared to the older adults in all the SSQ subscales (Speech, Spatial, and Qualities) and reported a lesser frequency of the problems in three of the four APHAB subscales (Ease of communication, Reverberation, and Background Noise). There was no significant difference in the frequency of the problems reported in the Aversiveness subscale. Self-reported normal-hearing listeners do not rate their listening ability at the top of the ability scale. Additionally, the large dataset presented here has a potential normative value for the SSQ and the APHAB questionnaires for self-reported normal-hearing adult listeners.

## 1. Introduction

Questionnaires are frequently used in audiologic clinics worldwide to assess listeners’ subjective sense of their listening ability in everyday complex listening scenarios. The Speech, Spatial, and Qualities of Hearing Scale (SSQ; [1]) and the Abbreviated Profile of Hearing Aid Benefit (APHAB; [2]) are two such instruments designed to measure an individual’s self-perception of their hearing abilities and difficulties perceived in various everyday listening conditions.

The Speech, Spatial, and Qualities of Hearing Scale (SSQ; [1]) is a 50-item questionnaire developed to assess listeners’ self-perception of their listening ability and listening experience in a variety of everyday complex listening situations that often involve spatial hearing. The questions probe into an individual’s auditory scene analysis and cognitive abilities and their relation to the perception of sound. One question was excluded as it pertained only to hearing aid uses. The 49-items used in the questionnaire are divided into three subscales: Speech Hearing, Spatial Hearing, and Qualities of Hearing. Individuals rate their ability or experience with each item on a scale of 0–10, where 10 indicates a high level of ability or experience with the item, and 0 indicates a low level of ability or experience with the item.

Investigators have been using the SSQ to obtain individuals’ self-perception of their hearing abilities in a variety of populations. Gatehouse and Noble [1] validated the SSQ using a large cohort of adults with hearing loss in unaided and aided conditions. Noble et al. [3] used the SSQ to measure self-rated disabilities among younger and older cochlear implant (CI) users and concluded that younger CI listeners had significantly higher SSQ scores than older CI listeners. Yawn et al. [4] used the SSQ in assessing the subjective improvements in adults with bilateral CIs. Their results indicated that the SSQ score in all the three subscales improved in the bilateral CI condition when compared to the preoperative bimodal condition. Dumper et al. [5] used the SSQ to measure the self-reported hearing abilities of individuals using bone-anchored hearing aids and showed that individuals with bilateral conductive hearing loss and unilateral conductive hearing loss showed a significantly higher preference for sound quality for aided speech compared to individuals with unilateral mixed hearing loss. Zahorik and Rothpletz [6] administered the SSQ to a large cohort of young normal-hearing listeners and provided normative data on each of the self-administered SSQ items and described the psychometric properties of the SSQ for the abovementioned population. Banh et al. [7] compared the SSQ results of younger and older individuals and concluded that older listeners with normal-hearing thresholds up to 4 kHz had significantly lower scores on all the three SSQ scales compared to younger listeners with normal hearing. Saunders et al. [8] used the SSQ to characterize self-reported hearing-related difficulties in a blast-exposed veteran population and concluded that the SSQ scores of younger blast exposed veterans were similar to the scores of older individuals with hearing loss, as reported by Gatehouse and Noble [1]. Moreover, Singh and Pichora-Fuller [9] examined the test–retest properties of the SSQ questionnaire and found a strong correlation in self-reported hearing abilities between the interview method and self-administered method, indicating that the self-administration of the SSQ questionnaire was effective and less time-consuming.

Over the last ten years, the SSQ has been translated and validated into several languages other than English with children and adults as participants, e.g., in Brazilian Portuguese with older adults [10], in Dutch with children and adolescents [11], in Dutch with younger and older adults [12], in Italian with children [13], in Korean with older adults [14], in Iranian with older adults [15], and in French with younger and older adults [16]. Moreover, the results reported from these various language versions indicate a good agreement [17], suggesting its potential use as an international standard for the self-reported measure of one’s hearing ability. Additionally, various short forms of the SSQ have been developed and validated, for both children and adults, which are suitable for using in audiological clinics, for instance, in younger adults [18]; in younger and older adults [19] and in children, younger, and older adults [20].

The Abbreviated Profile of Hearing Aid Benefit (APHAB; [2]) is a 24-item self-assessment questionnaire in which listeners report the level of difficulty they experience while communicating in various everyday listening situations. The APHAB questions are divided into four subscales: Ease of Communication, Reverberation, Background Noise, and Aversiveness. Individuals rate their experience with each item by selecting one of seven response options that range in frequency from never (1%), to always (99%).

Even though the APHAB was initially developed to quantify the disability associated with hearing loss and the reduction in the abovementioned disability that happens with the uptake of a hearing aid, it has been used to obtain a self-perception of hearing abilities in individuals with normal hearing, cochlear implant users, and bone-anchored hearing aid users. Löhler et al. [21] used listeners with normal hearing and listeners with hearing loss to determine the diagnostic value of the APHAB questionnaire and concluded that hearing loss at 25 dB had an influence on the APHAB scores. Linstrom et al. [22] observed subjective short-term and long-term benefits for bone-anchored hearing aid users in the three subscales (EC, BN, and RV), but not in the AV subscale. García et al. [23] investigated the communicative benefits in adult patients with single-sided deafness using the APHAB and concluded that the subscales scored improved post-implantation in the three subscales (EC, BN, and RV), but not in the AV subscale. Duret et al. [24] showed similar results to García et al. [23] in a sample of bimodal CI (CI in one ear and a hearing aid in the other ear) listeners. Over the last ten years, the APHAB has been translated and validated into several languages other than English, e.g., Swedish [25], Norwegian [26], German [27], and Korean [28]. Moreover, the Hearing Aid Research Laboratory at the University of Memphis has the APHAB questionnaire in more than 20 languages, and it is currently being developed in three more languages [29]. Dornhoffer et al. [30] compared the traditional audiologic and patient-reported measures of aided performance and concluded that the hearing aid benefit assessed with audiologic measures were poor predictors of patient-reported benefits. de Andrade et al. [31] used the APHAB to investigate the quality of life in listeners with mild to moderate sensorineural hearing loss and concluded that these individuals could perceive a reduced quality of life, thereby limiting their participation in everyday activities.

Although both the SSQ and the APHAB measure individuals’ self-reported hearing ability, there are few studies in the literature that compare the outcomes of these two questionnaires on the same group of listeners. Valente et al. [32] used the SSQ and APHAB to investigate whether there were any significant differences in the subjective scores using the manufacturer’s first-fit and hearing aids programmed-fit to the National Acoustics Laboratories Nonlinear Version 2 (NAL-NL2) prescriptive targets using real ear measures. Dillon et al. [33] used the APHAB and SSQ to study the improvement in individuals’ quality of life before and after their implantation on cochlear implant listeners. However, both these studies did not investigate the relationship between the two questionnaires. The goal of this study is to compare the responses to the SSQ and the APHAB from a large cohort of self-reported normal-hearing adult listeners and evaluate the relationship between the SSQ subscales and the APHAB subscales.

## 2. Materials and Methods

### 2.1. Participants

A total of 136 younger listeners (YL; mean age = 22.6 years, range: 19–30 years) and 118 older listeners (OL; mean age = 53.7 years, range: 40–70 years) participated in this study. All YLs received course credit for their participation and data were collected using Qualtrics software. The OLs data were collected using a combination of Qualtrics software and a traditional pen-and-paper method. All participants completed a series of intake questions, and those that responded “yes” to questions related to having hearing loss or being a hearing aid user were excluded from the study. All younger listeners were provided with course credit for one of their courses for participating in this research. The older listeners were not provided any compensation for their effort.

### 2.2. Materials

Each participant completed a 49-item SSQ [1], and a 24-item APHAB [2] questionnaire either via Qualtrics software or a traditional pen-and-paper method. The order of the questionnaires among the participants was randomized.

## 3. Data Analysis

Analyses were performed with SPSS 25.0 (IBM Corp., Armonk, NY, USA). A mixed ANOVA was used to investigate the effects of age on the various subscales for both questionnaires. Pearson’s correlations were computed to examine the relationships between the subscales.

## 4. Results

**Figure 1:**
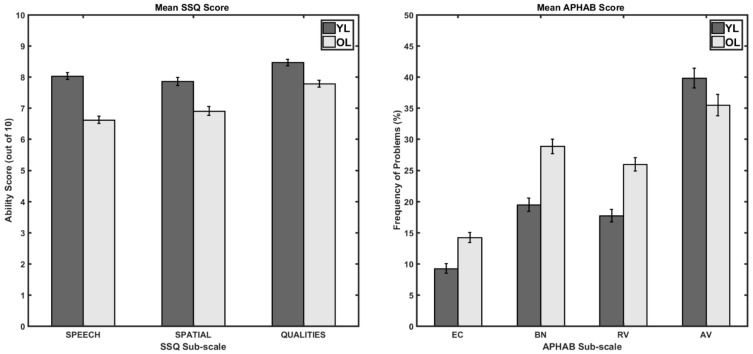
The left panel shows the average ability scores for the three SSQ subscales and the right panel shows the average percentage of problems encountered for the four APHAB subscales (EC: Ease of Communication; BN: Background Noise; RV: Reverberation; AV: Aversiveness) for the younger (darker bars) and older (lighter bars) listeners. The error bars indicate ±1 SEM. Note that more hearing difficulties are indicated by smaller and taller bars in the right and left panels, respectively.

Table 1 shows the descriptive statistics for the mean ratings in the subscales of the SSQ and APHAB questionnaires. The table also shows the results of the simple effect analyses comparing the mean scores between younger and older listener groups on the various subscales of the two questionnaires. The left panel of Figure 1 shows the mean ratings in the three subscales for the SSQ questionnaire, and the right panel shows the mean percent of the problems in the four subscales for the APHAB questionnaire. A mixed ANOVA revealed a significant main effect of the age group (younger and older) (SSQ: *F*(1, 252) = 77.5, *p* < 0.001, $\eta_{p}^{2}$ = 0.24; APHAB: *F*(1, 252) = 16.32, *p* < 0.001, $\eta_{p}^{2}$ = 0.06) and a significant interaction between the age group and the subscales (SSQ: *F*(2, 504) = 5.79, *p* = 0.02, $\eta_{p}^{2}$ = 0.03; APHAB: *F*(3, 756) = 18.56, *p* < 0.001, $\eta_{p}^{2}$ = 0.07). Simple effects analysis (the *F* values, degrees of freedom and the corresponding *p* values are reported in Table 1) revealed younger listeners had significantly higher self-perceived hearing abilities than the older adults in all the three SSQ subscales, and younger listeners had significantly fewer frequency of problems than the older adults in all the Ease of Communication, Reverberation, and Background Noise APHAB subscales. There was no significant difference in the frequency of problems reported for the aversiveness APHAB subscale between the younger and older adults (*p* = 0.06). 

**Figure 2:**
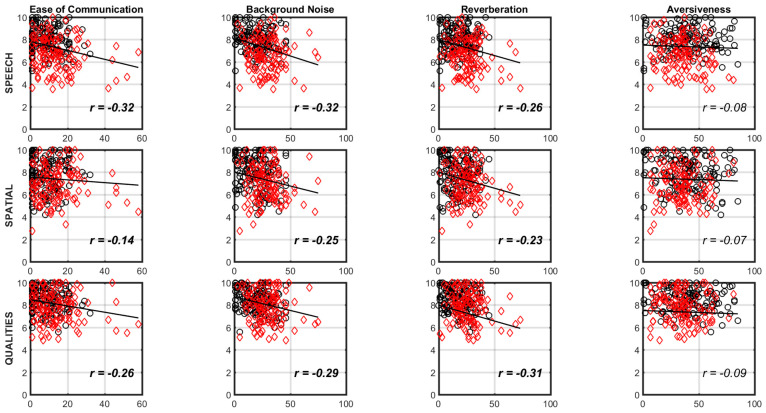
The top row shows the scatterplot between the speech SSQ subscale and the four APHAB subscales, the middle row shows the scatterplot between the spatial SSQ subscale and the four APHAB subscales, the bottom row shows the scatterplot between the qualities SSQ subscale and the four APHAB subscales. The x-axis represents scores from the subscales of the APHAB questionnaire while the y-axis represents scores from the subscales of the SSQ questionnaire. The open black circles indicate data from younger listeners, while the open red diamonds indicate data from older listeners in all the panels. The solid black line inside the panel shows the best fit line for the data. Bold correlations inside the panels are significant at *p* < 0.05.

A correlation analysis was conducted to investigate the relationship between the subscales of the two questionnaires used in this study. Figure 2 shows the scatter plot between the various subscales of the questionnaires. As indicated in Figure 2, all the three SSQ subscales were significantly negatively correlated with each of the Ease of Communication, Reverberation, and Background Noise APHAB subscales. However, there was no significant relationship between the aversiveness APHAB subscale and the three SSQ subscales.

## 5. Discussion

The main purpose of this study was to investigate the effects of age on the self-perceived hearing abilities and to study the relationships between the subscales of the SSQ and APHAB questionnaires in a large sample of self-reported normal-hearing listeners. As expected, younger listeners (YL) reported higher abilities to communicate in complex listening environments compared to older listeners (OL) when questioned using the SSQ. However, it is interesting to note that both the groups still perceived some problems in such environments. Both YL and OL reported the highest ability in the qualities subscale. However, the YL reported the lowest ability in the spatial subscale, whereas the OL reported the lowest ability in the speech subscale. Additionally, the average subscale scores and the order of the scores (spatial < speech < qualities) reported in this study for the YL group were similar to the scored reported in the literature with audiometrically screened normal-hearing (≤20 dB HL) listeners [6,34] and the average subscale scores and the order of the scores (speech < spatial < qualities) reported in this study for the OL group were similar to the scored reported in the literature [7]. Additionally, the interaction patterns presented here were similar to the patterns reported in [7]. On the other hand, the average subscale scores reported in [34] for the OL group was higher compared to the average scores obtained in this study. However, it should be noted that Fullgrabe et al.’s [34] study was limited by their sample size (*n* = 21; mean age = 67 years, age range = 60–79 years), compared to this study, which has a much larger sample size. Additionally, this difference in the study sample could have led to the absence of age and subscale x age interaction effects in [34].

For the APHAB questionnaire, YL had a significantly lower frequency of problems for the EC, BN, and RV subscales and there was no significant difference in the frequency of problems for the AV subscale. The mean frequency of problems reported for both the groups reported here were larger than the scores reported in [34]. However, as indicated earlier, it should be noted that Fullgrabe et al.’s study [34] had fewer participants compared to this study.

Significant negative correlation between the three SSQ subscales and Ease of Communication, Reverberation, and Background Noise APHAB subscales indicated that individuals who perceived higher abilities in the SSQ subscales reported a lower frequency of problems in the three APHAB subscales. The fourth APHAB subscale, aversiveness, was not significantly correlated with any of the SSQ subscales. This was expected as the underlying construct of the aversiveness subscale was to assess negative reactions to environmental sound and how noisy situations were misperceived [2,21,35] which is different from the underlying constructs of the SSQ subscales which assess the ability to hear speech in a variety of competing contexts, the ability to use binaural cues in spatial hearing, the ability to segregate multiple streams of sounds, and the ability to attend simultaneous speech streams [1].

Even though the SSQ subscales and three of the four APHAB subscales are correlated, the amount of variance accounted for by these comparisons ranged from 0.02 to 0.10. Given the fact that the variance accounted is small, it would be beneficial to use both the questionnaires in the evaluation of hearing-impaired subjects with any type of hearing aid. If the clinician wishes to factor in listener fatigue and the time taken to complete the questionnaires, the responses could be collected before the clinical appointment using a self-administered method (questionnaires mailed or creating a web-based system). Alternatively, a new questionnaire could be created incorporating the best aspects of both the SSQ and the APHAB questionnaires. However, the Aversiveness subscale of the APHAB should certainly be considered while developing this new questionnaire as it provides unique and very important information that is not provided by the SSQ questionnaire or the other three subscales of the APHAB questionnaire.

Overall, the results presented here indicate that both the SSQ and APHAB probe self-perceived hearing abilities for similar circumstances, except for the fact that the aversiveness subscale of the APHAB probes an entirely different dimension which is not probed by any of the SSQ subscales. Additionally, self-reported normal-hearing younger listeners do not necessarily rate their listening abilities at the top of the ability scale in all three SSQ subscales. The big limitation of this study is that the data presented here are from a large cohort of self-reported normal-hearing participants and the audiometric thresholds are not measured. The absence of the audiometric thresholds from the participants should be taken into consideration while interpreting these results as the presence of high-frequency hearing loss or slight/mild hearing loss in the older listeners group could have impacted the scores in the two questionnaires. Together, the results presented here suggest that this dataset has a potential normative value, and more data need to be collected, especially from middle-aged participants, to develop normative data as a function of age group.

## Figures and Tables

**Table 1:** Descriptive statistics for the mean ratings of various subscales of the SSQ and APHAB questionnaire and the results of simple effect analyses comparing the mean scores between the younger and older listeners.

Questionnaire	Sub Scale	Younger Listeners				Older Listeners				Simple Effect Analysis	
		*M*	*SD*	95% CI		*M*	*SD*	95% CI			
				*LB*	*UB*			*LB*	*UB*	*F* (1, 252)	*p*
SSQ	SPCH	8.02	1.11	7.83	8.21	6.62	1.49	6.35	6.89	73.61	<0.001
	SPAL	7.86	1.46	7.62	8.11	6.91	1.56	6.63	7.19	25.03	<0.001
	QUAL	8.47	1.03	8.31	8.64	7.77	1.37	7.52	8.02	20.39	<0.001
APHAB	EC	9.22	7.14	8.02	10.43	14.2	10.79	12.23	16.17	19.22	<0.001
	BN	19.45	11.96	17.42	21.48	28.3	13.12	25.91	30.69	35.43	<0.001
	RV	17.72	10.58	15.93	19.51	25.96	12.97	23.59	28.32	31.05	<0.001
	AV	39.79	20.29	36.35	43.23	35.46	16.07	32.53	38.39	3.46	0.064

Note. CI = confidence interval, *LB* = lower bound, *UB* = upper bound. SPCH denotes Speech, SPAL denotes Spatial, and QUAL denotes Quality subscales of the SSQ questionnaire. EC denotes Ease of communication, BN denotes Background Noise, RV denotes Reverberation, and AV denotes Aversiveness subscales of the APHAB questionnaire. The *p* values were adjusted for multiple comparisons (Bonferroni correction).

## Data Availability

The data that support the findings of this study are available from the corresponding author, Nirmal Srinivasan, upon reasonable request.
